# Diversity and the Origin of *Perlodinella* Klapálek 1912 (Plecoptera: Perlodidae) in Qinghai Province, China

**DOI:** 10.3390/insects16050520

**Published:** 2025-05-14

**Authors:** Qing-Bo Huo, Shi-Xiong Fan, Ya-Fei Zhu, Yu-Zhou Du

**Affiliations:** 1 College of Plant Protection & Institute of Applied Entomology, Yangzhou University, Yangzhou 225009, China; jw30th@163.com (Q.-B.H.); sxfan06@163.com (S.-X.F.); z1525747447@139.com (Y.-F.Z.); 2 Jiangsu Province Engineering Research Center of Green Pesticides, Yangzhou University, Yangzhou 225009, China

**Keywords:** stonefly, taxonomy, species diversity, larva, COI, biogeography

## Abstract

The article presents integrative research of the perlodid genus *Perlodinella* in Qinghai Province, northwestern China. *P. tatunga* Wu, 1973 is considered a junior synonym of *P. kozlovi* Klapálek, 1912, with a further description of intraspecific morphological variability, while *P. unimacula* Klapálek, 1912 is considered to be nomen dubium. The COI barcodes of the three valid species in Qinghai, *P. epiproctalis* (Zwick, 1997), *P. kozlovi* Klapálek, 1912, and *P. microlobata* Wu, 1938 are firstly sequenced, enabling adult–larva matching and the analysis of genetic diversity. The larval morphology of *P. kozlovi* and *P. microlobata* is described for the first time. Additionally, the biology, ecological adaptability, and fungal infections of *Perlodinella* are firstly recorded with an environment-related comparison. The discussion of the origin and immigration of the genus is also provided.

## Simple Summary

As the highest-altitude region on average in the world, the Qinghai-Tibet Plateau harbors the remarkable fauna of stoneflies. *Perlodinella* is endemic to China and one of the most common perlodids in Qinghai, yet it remains poorly understood by scientists. This study supplements the taxonomic, biological, and ecological knowledge of *Perlodinella* in Qinghai based on morphology, molecular biology, and extensive field observations, while also providing new insights into the biogeography of this genus.

## 1. Introduction

*Perlodinella* Klapalek, 1912 is a rare genus of the stonefly family Perlodidae, with only eleven species known from China, including Hebei, Heilongjiang, Hubei, Gansu, Liaoning, Neimenggu (Inner Mongolia), Qinghai, Shanxi, and Xizang (Tibet) [1,2,3,4,5,6,7,8,9,10,11,12]. Qinghai Province is located in the northwest of China, bordering Gansu, Sichuan, Xizang, and Xinjiang. Previously, five *Perlodinella* species (*P. epiproctalis* (Zwick, 1997), *P. kozlovi* Klapalek, 1912, *P. microlobata* Wu, 1938, *P. tatunga* Wu, 1973, and *P. unimacula* Klapalek, 1912) have been recorded in Qinghai, which currently presents as the province with the highest species diversity for this genus. However, the *Perlodinella* from Qinghai has rarely been studied since its proposal. Recently, only *P. epiproctalis*, *P. kozlovi*, and *P. microlobata* have been further revised and redescribed, while *Rauserodes* Zwick, 1999 has been considered a synonym of *Perlodinella* [9,10,13,14]. Now, there are still two species (*P. tatunga* and *P. unimacula*) remaining in Qinghai, with their taxonomic status in doubt because of a lack of a clear morphological diagnosis over several decades.

During the investigation and sampling in Qinghai, 2024, we returned to Guanghui Temple, Datong County, the type locality of *P. tatunga*. Plenty of adults, larvae, exuviae, and eggs of *Perlodinella* were collected from the only river around the temple. After examination, we found that there is only one *Perlodinella* species in this area. The morphology, distribution, and emergence date of adults are consistent with the original record of *P. tatunga* [3]. Therefore, we consider that these specimens are *P. tatunga*. However, we also found that the morphology of *P. tatunga* is exactly the same as *P. kozlovi*, so we have to consider *P. tatunga* is a junior synonym of the latter. Additionally, *P. unimacula* is still questionable in taxonomy because its type specimen consists of only one female, lacking a detailed description, and it has been unavailable for study. The type locality of *P. unimacula* overlaps with the distribution of *P. kozlovi* and *P. microlobata*, and the diagnosis of female *P. unimacula* is very similar and unidentifiable to several congeners (*P. kozlovi*, *P. microlobata* and *P. tibetensis* Huo & Du, 2022). In this study, we regard *P. unimacula* as a new nomen dubium.

At present, preliminary progress has been made in the genetic study of Chinese stoneflies based on DNA barcoding (mainly based on the COI gene), but there are still few related works known [15,16,17,18]. The available COI barcodes of *Perlodinella* are scarce, which restricts species identification and adult–larva matching. In this study, the COI gene fragments of *P. epiproctalis*, *P. kozlovi*, and *P. microlobata* from different regions of Qinghai were sequenced for the first time. The matching of female/male adults and larvae of *P. kozlovi* and *P. microlobata* was conducted, with a comparison of the intraspecific and interspecific genetic distances discussed. Thus, the morphology of larvae (or exuviae) of the two species is described for the first time on the basis of molecular identification. Before this study, only one species (*P. epiproctalis*) had a description based on the larval morphology [9].

Moreover, the biology of *Perlodinella* species in Qinghai was only briefly documented in Huo et al. [8,9,10]. Our study provides further biological observations and records the habitat preferences and behaviors of the three *Perlodinella* species in Qinghai. Their negative phototaxis (particularly the semi-fossorial habits of *P. epiproctalis*) is discussed, and the fungal infection in *P. kozlovi* and *P. microlobata* is firstly reported to science. The emergence date of *P. kozlovi* adults in environments at different altitudes has also been confirmed to vary significantly. Finally, the distribution of multiple populations of *P. epiproctalis*, *P. kozlovi*, and *P. microlobata* in Qinghai and surrounding areas are updated. Based on the previous biogeographical study of stoneflies [18,19,20], we offer new insight into the origin, immigration, and distributive limitations of *Perlodinella* based on the orientation of mountains and flow directions of water systems of western China.

## 2. Materials and Methods

### 2.1. Filed Observation and Specimen Preparation

Specimens were collected by hand and preserved in 75% and 100% ethanol. Photographs were taken with a PowerShot SX730 HS camera (Canon, Tokyo, Japan) and the KEYENCE VHX-5000 system (Keyence, Ōsaka, Japan) and subsequently optimized in Adobe Photoshop CS6. The type materials have been deposited into the Insect Collection of Yangzhou University (ICYZU), Yangzhou, Jiangsu Province, China.

### 2.2. DNA Extraction, Amplification, and Sequencing

The specimens of *P. epiproctalis*, *P. kozlovi*, and *P. microlobata* used for molecular analyses were barcoded from 18 individuals (COI fragments, 658 bp) collected from 19 individuals from different localities of Qinghai (Table 1). Genomic DNA was extracted using the FastPure Cell/Tissue DNA Isolation Mini Kit (Vazyme Biotech, Nanjing, China), following the manufacturer’s instruction. The DNA barcode region of the mitochondrial COI gene was amplified using the primers COI4-F (5-TGTAAAACGACGGCCAGTAAACTAATARCCTTCAAAG-3) and LepR1 (5-TAAACTTCTGGATGTCCAAAAAATCA-3) [21,22] in a 25 µL reaction volume, containing 12.5 µL of 2× Taq Master Mix (Vazyme Biotech, Nanjing, China), 1 µL of each primer, 2 µL of DNA template, and 8.5 µL of ddH_2_O. The polymerase chain reaction (PCR) amplification was conducted under the following conditions: 95 °C for 30 s, 40 cycles of 95 °C for 10 s, 52 °C for 50 s, 65 °C for 1 min, and final elongation at 65 °C for 10 min. The PCR products were confirmed via electrophoresis in 1.0% agarose gels and then sequenced bidirectionally by Tsingke BioTech Co., Ltd., Nanjing, China.

The DNA barcodes were assembled based on the reference sequence using GENEIOUS v. 9.0 [23]. Sequences amplified using primers MtRWF1 and LepR1 contain the tRNA-W region, and the sequence of the standard DNA barcode region of *Perlodes microcephalus* (GenBank accession number: KY262014.1) was selected to join in the multiple sequence alignment in MEGAv.11.0 [24] to confirm that the tRNA-W region was completely trimmed. The sequences (Table 1) were submitted to National Center for Biotechnology Information (NCBI).

### 2.3. Genetic Analyses

K2P genetic distances were calculated using MEGA v. 11.0. The maximum likelihood (ML) analysis was computed with IQ-Tree v. 1.6.12 (http://www.iqtree.org/, accessed on 9 January 2025), and the data were transformed into phylogenetic trees using FigTree v. 1.4.2 (http://tree.bio.ed.ac.uk/software/figtree/, accessed on 9 January 2025). The parameters of ML analysis are as follows: test of phylogeny, bootstrap method with 1000 bootstrap replications; substitution type, nucleotide; model/method, Kimura 2-parameter model; rates among sites, uniform rates; gaps/missing data treatment, complete deletion; ML heuristic method, nearest-neighbor-interchange (NNI); initial tree for ML, make initial tree automatically (Default-NJ/BioNJ); branch swap filter, none. *Nemoura geei* Wu, 1929 (GenBank accession number: MK132385.1) was used as the outgroup in ML analyses.

## 3. Results and Discussion

### 3.1. Genetic Diversity and Species Identification Based on COI Sequences

**Figure 1:**
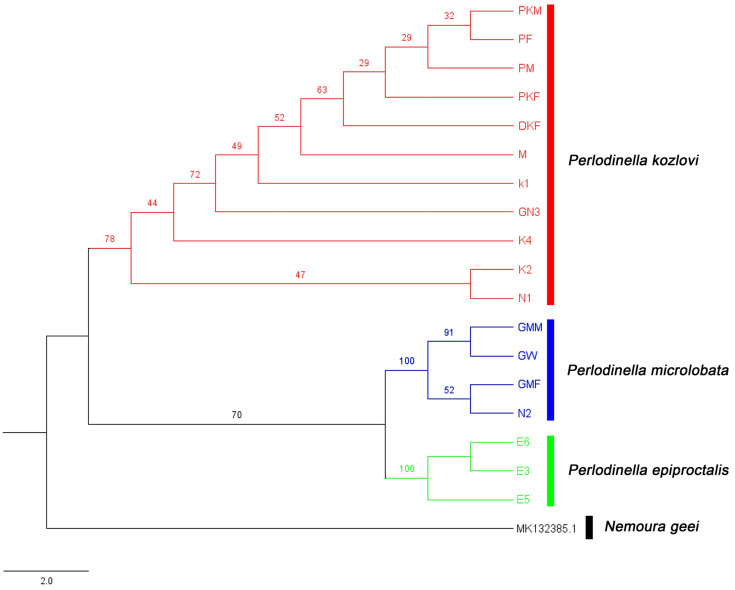
Phylogenetic trees of COI gene. Phylogenetic relationships among three species of *Perlodinella* based on a maximum likelihood (ML) analysis (numbers at the nodes are ML bootstrap values; the scale bar represents the rate of base substitutions).

According to the results of the phylogenetic analysis, the monophyly of the three *Perlodinella* species and the identity of these unknown larvae have been well confirmed (Figure 1, Table S1). The intraspecific genetic distances among *P. epiproctalis*, *P. kozlovi*, and *P. microlobata* are no more than 1.4%, while the interspecific distances are no more than 7% (Table S1). These results align with the general 2% threshold for interspecific divergence. However, Chen [16] and Yang and Du [17] have both demonstrated that the genetic differences within and between species in the order Plecoptera are significantly higher than this value, especially among different geographical populations. The most likely explanation for the findings of this study is the limited number of sequences used and the potential for substantial variation in genetic characteristics among families within Plecoptera. Further sampling and sequencing will be necessary in the future to explore the maximum intraspecific and interspecific genetic distances of these species.

The genetic distances and phylogenetic analysis support the preliminary morphological identification results, particularly confirming that the method used by Huo et al. [10] to distinguish *P. microlobata* and *P. kozlovi* from other closely related species based on the morphology of the female subgenital plate is reliable. Additionally, since the larvae of *P. kozlovi* and *P. microlobata* had not been previously described, it was unclear to which species these specimens belonged. Based on the COI sequences, this study successfully matched the larvae and adults of *P. kozlovi* and *P. microlobata*, providing a foundation for supplementary morphological descriptions of the larvae.

### 3.2. Species Diversity and the Intraspecific Morphological Variability

**Figure 2:**
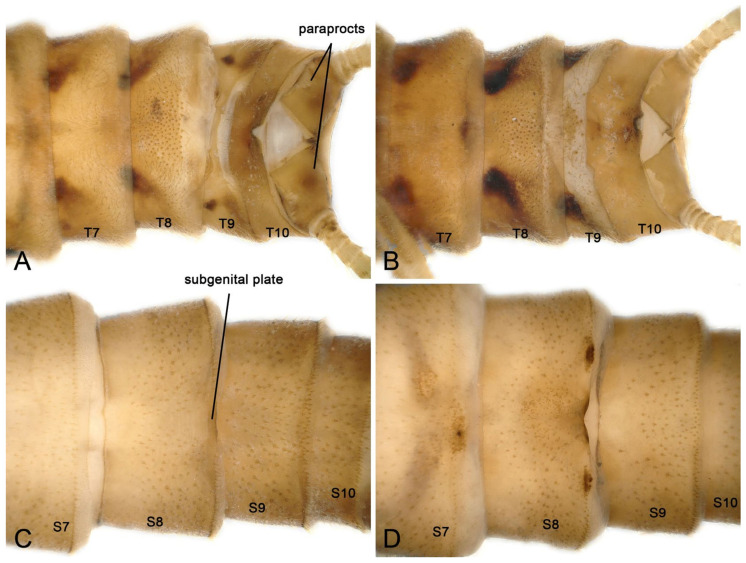
*Perlodinella epiproctalis* from Qilian Mountains. (**A**,**B**) Male terminalia without/with sclerotized on tergum 9, dorsal views; (**C**,**D**) female larvae without/with more sclerotized and darker pigmentation on the sternum 8, ventral views.

According to our research, *Perlodinella* in Qinghai currently contains only three valid species. Tianjun County and Tongde County are the regions with the highest diversity of the genus, with two species recorded each. However, only *P. kozlovi* and *P. microlobata* have overlapping distributions, and there is no area where all three species have been recorded at the same time. The species list, supplementary descriptions, and distributive information are as follows:

***Perlodinella epiproctalis* (Zwick, 1997) (Figure 2)**

*Rauserella epiproctalis* Zwick, 1997: 489.

*Rauserodes epiproctalis* Zwick, 1999: 168.

*Perlodinella epiproctalis*: Huo et al., 2022: 390.

Material examined: The same collection sites recorded in Huo et al. [8,9]: 10 males, 4 females, China, Qinghai Province, Haibei Tibetan Autonomous Prefecture, Menyuan Hui Autonomous County, Liuhuanggou, 3720 m, 101°21′16″ E, 37°46′22″ N, 2019-VIII-14, leg. Wang Lu-Yu, Chen Zhen-Ning: 4 males, 2 females, Liuhuanggou, 101°18′8″ E, 37°41′56″ N, 2019-VII-29, leg. Kong Xiang-Bo, Fang Jia-Xing, He Yun-Chuan: 8 males, 4 females, 10 nymphs, China, Qinghai Province, Haibei Tibetan Autonomous Prefecture, Menyuan Hui Autonomous County, Laohugou, 3549 m, 101.582778° E, 37.585556° N, 2021-VII-4, leg. Huo Qing-Bo, Zang Hao-Ming, Shen Rong-Rong; 11 males, 9 females, 8 nymphs, Qinghai Province, Qilian County, Longkong, 3425 m, 100.680833° E, 38.067500° N, 2021-VII-5, leg. Huo Qing-Bo, Zang Hao-Ming; all specimens disposed in ICYZU.

Distribution: China: Qinghai (Menyuan, Qilian, Wulan counties).

Supplementary description for adult and larva: The male tergum 9 posteriorly membranous, bearing sensilla basiconica of two types, separate or connected by irregular sclerites on the anterior or posterior of the membranous area (Figure 2A,B). Tergum 10 with a pair of dark lateral spots, and a dark longitudinal median band, sometimes lacking not; posterior margin heavily sclerotized, curved upward and fully covered with sensilla basiconica (Figure 2A,B), by Huo et al. [8]. The mature female larva sometimes has more sclerotized and darker pigmentation (Figure 2C,D) around the subgenital plate (posterior margin on sternum 8).

***Perlodinella kozlovi* Klapálek, 1912 (Figure 3, Figure 4, Figure 5, Figure 6, Figure 7, Figure 8, Figure 9 and Figure 10)**

*Perlodinella kozlovi* Klapálek, 1912: 28.

*Perlodinella tatunga* Wu, 1973: 106. **(syn. nov.)**

Material examined: 26 males, 34 females, c.a. 20 exuviae, China, Qinghai Province, Xining City, Datong Hui and Tu Autonomous County, Dongxia Town, S20 highway, south Guanghui Temple, 2024-V-27, 37.023000° N, 101.783832° E, 2575 m. Leg. Huo Qing-Bo, Fan Shi-Xiong; 15 males, 19 females, Qinghai Province, Xining City, Datong Hui and Tu Autonomous County, Dongxia Town, Dongxia Overpass, north Guanghui Temple, 2024-V-27, 37.023000° N, 101.783832° E, 2575 m. Leg. Huo Qing-Bo, Fan Shi-Xiong; 10 males, 12 females, 5 exuviae, Qinghai Province, Xining City, Datong Hui and Tu Autonomous County, Xinzhuang Town, G227 highway, Baoku River, 2024-V-28, 37.067971~37.245938° N, 101.574172~101.460556° E, 2547~2855 m. Leg. Huo Qing-Bo, Fan Shi-Xiong; 2 larvae, Qinghai Province, Hainan Tibetan Autonomous Prefecture, Xinghai County, Ziketan Town, Ziqing Road, Ehegan, 2024-V-30, 35.581647° N, 99.863318° E, 3004 m. Leg. Huo Qing-Bo, Fan Shi-Xiong; 8 males, 12 females, 6 larvae, c.a. 20 exuviae, Qinghai Province, Hainan Tibetan Autonomous Prefecture, Tongde County, Zhihoumai Village, Gagan Reservoir, 2024-VI-4, 35.123291° N, 100.622657° E, 3347 m. Leg. Huo Qing-Bo, Fan Shi-Xiong; c.a. 4 exuviae, Qinghai Province, Huangnan Tibetan Autonomous Prefecture, Tongren City, G213 highway, Longwu River, 2024-VI-5, 35.379444° N, 101.959975° E, 2675 m. Leg. Huo Qing-Bo, Fan Shi-Xiong; all specimens disposed in ICYZU. The same collection sites recorded in Huo et al. (2022) Lectotype male (present designation), paralectotypes 1 male, 2 females, China, Sichuan Province, Kham area, Yangtze River Basin, Yalong River, approximately 33° N, 98° E), 1901-IV, leg. P. K. Kozlov (NMP) [9]; 18 males, 25 females, China, Qinghai Province, Haixi Mongol and Tibetan Autonomous Prefecture, Tianjun County, 2021-VII-13, 37.56869° N, 98.65726° E, 3545 m. Leg. Huo Qing-Bo, Zang Hao-Ming, Wang Ya-Meng, Sun Chen-Tao, Li Cong (ICYZU).

Distribution: China: Qinghai (Datong, Tianjun, Tongde, Xinghai counties, Tongren City), Gansu (Yuzhong County), Sichuan (Shiqu County).

**Figure 3:**
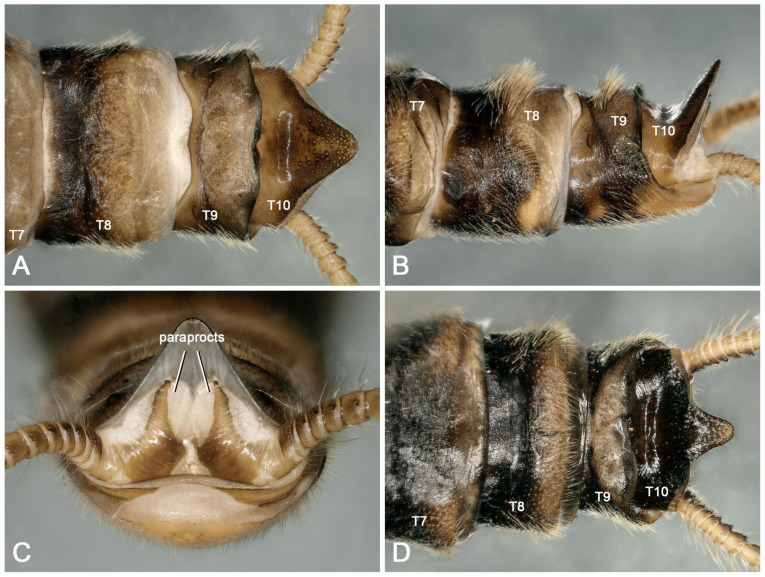
*Perlodinella kozlovi*, two males. (**A**–**C**) From Datong, Guanghui Temple, dorsal, lateral and caudal views; (**D**) from Tongde, the individual is more sclerotized with darker pigmentation, dorsal view.

**Figure 4:**
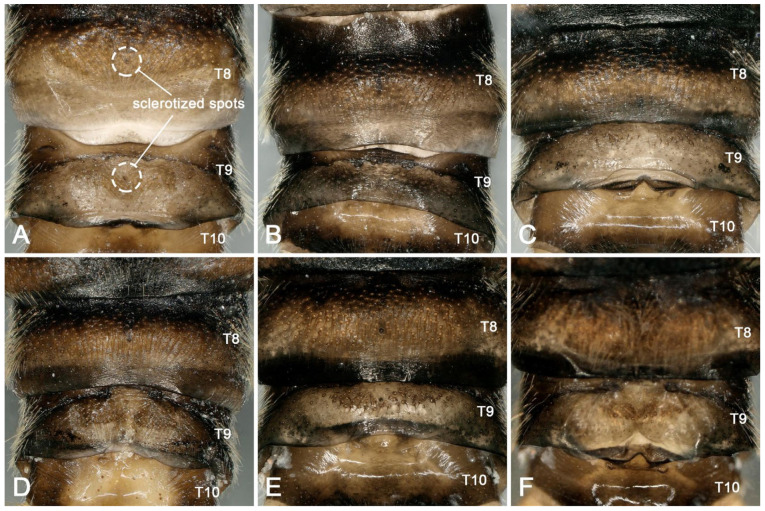
*Perlodinella kozlovi*, the sclerotized round spot in the middian terga 7 and 8 for the six males. (**A**–**C**) from Datong; (**D**) from Tongde; (**E**,**F**) from Tianjun.

Supplementary description for adult: Similar to individuals in other regions, this species shows intraspecific changes in pigment deposition (Figure 3A–D). In addition, we first discover that males of the species often have a dark, sclerotized round spot in the middle of terga 7 and 8 (Figure 4A–F). This feature was not observed in the type materials, and it was evidently overlooked in Huo et al. [9]. After re-examining the specimens collected in Tianjun and Datong, we found that the sclerotized spots do not appear to have any specific biological function and are sometimes absent (or only absent at tergum 8) in some individuals (Figure 4B–D,F). Therefore, these sclerotized spots are considered another normal intraspecific variation besides the variable color patterns on the head and pronotum.

**Figure 5:**
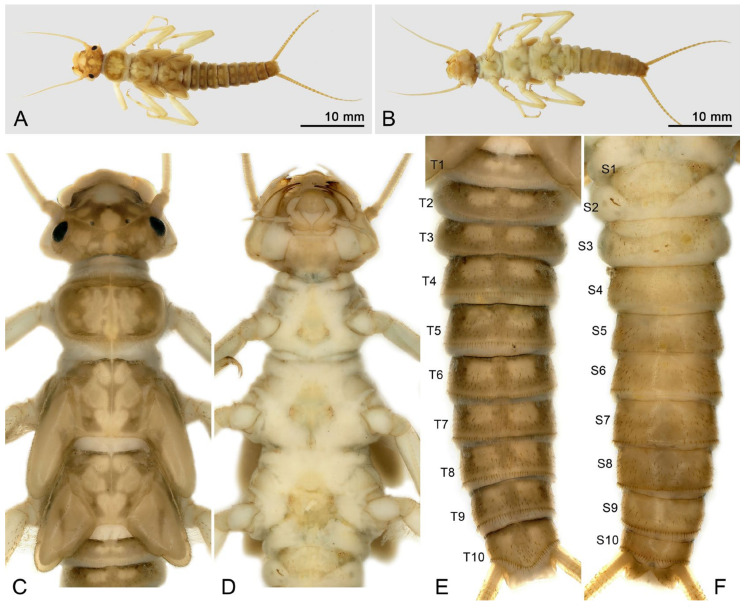
*Perlodinella kozlovi*, male larva from Datong, Guanghui Temple. (**A**,**B**) Dorsal and ventral habitus; (**C**,**D**) head and thoraxes, dorsal and ventral views; (**E**,**F**) abdominal segments, dorsal and ventral views.

**Figure 6:**
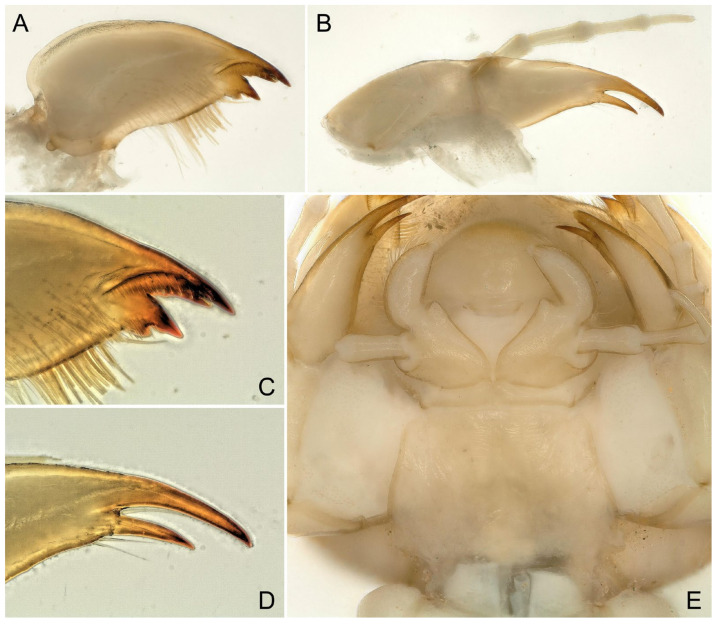
*Perlodinella kozlovi*, mouthpart of the male larva from Datong, Guanghui Temple. (**A**,**B**) Right mandible and lacinia, ventral views; (**C**,**D**) the teeth and setae of right mandible and lacinia, High Dynamic Range versions; (**E**) labium.

**Figure 7:**
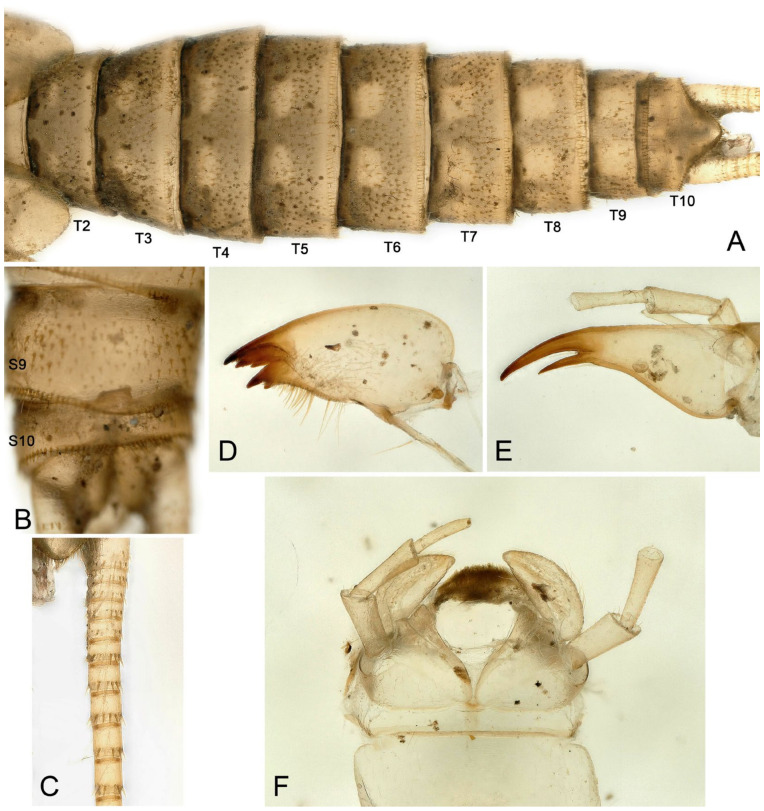
*Perlodinella kozlovi*, male exuviae from Datong, Guanghui Temple. (**A**) Abdominal segments, dorsal view; (**B**) sterna 9–10, ventral view; (**C**) cercus; (**D**,**E**) left mandible and lacinia, ventral views; (**F**) labium.

Larva: Mature male larvae/exuviae body length c.a. 30~32 mm (n = 5). Dorsum gray to olive green with some pale patterning; ventrally paler grey. Body covered by short colorless hairs (Figure 5, Figure 6 and Figure 7). Head with a large pale spot in front of the anterior ocellus extending onto the clypeus; M-line indistinct; interocellar area brown with small pentagonal, central pale spot, closed posteriorly; occiput with a pair of oval pale spots around the epicranial stem bordered by sinuate brownish rows behind each eye (Figure 5C). Submental gills are short and rounded (Figure 5D). Mandible bidentate, with 2 apical teeth and 3 subapical teeth at right, while 3 apical teeth and 2 subapical teeth at left (a mosaic structure); a remarkable line of c.a. 13 teeth occurred under the apical teeth; a patch of 15–18 hairs present ventral to the subapical teeth (Figure 6 and Figure 7). Lacinia bidentate, apically narrow, basal half expanded; 3–4 setae at the juncture of the apical teeth (Figure 6 and Figure 7). Paraglossae flat and fingerlike; glossa wide and semicircular, apex slightly truncated, with thick fine hairs (Figure 6 and Figure 7). Thoracic segments rectangular with wide, pale, median stripes; the pale stripes on pronotum almost a complete patch, but distinctly separate into 5~7 or more distinct blocks on the mesothorax and metathorax. Male wingpads long. Legs with few scattered brown spinules on surface (Figure 5C). Abdominal terga with two rows of large, oval, pale paramedial spots; spots are indistinct on tergum 10; tergum 10 with a small square extending on the median anterior margin (Figure 7B); all abdominal segments have a few short, stout spinules; paraprocts short, apex rounded (Figure 5E,F). Cerci with dorsal fringe of fine, silky, colorless hairs; each cercal segment with an apical whorl of short brown setae (Figure 7C).

**Figure 8:**
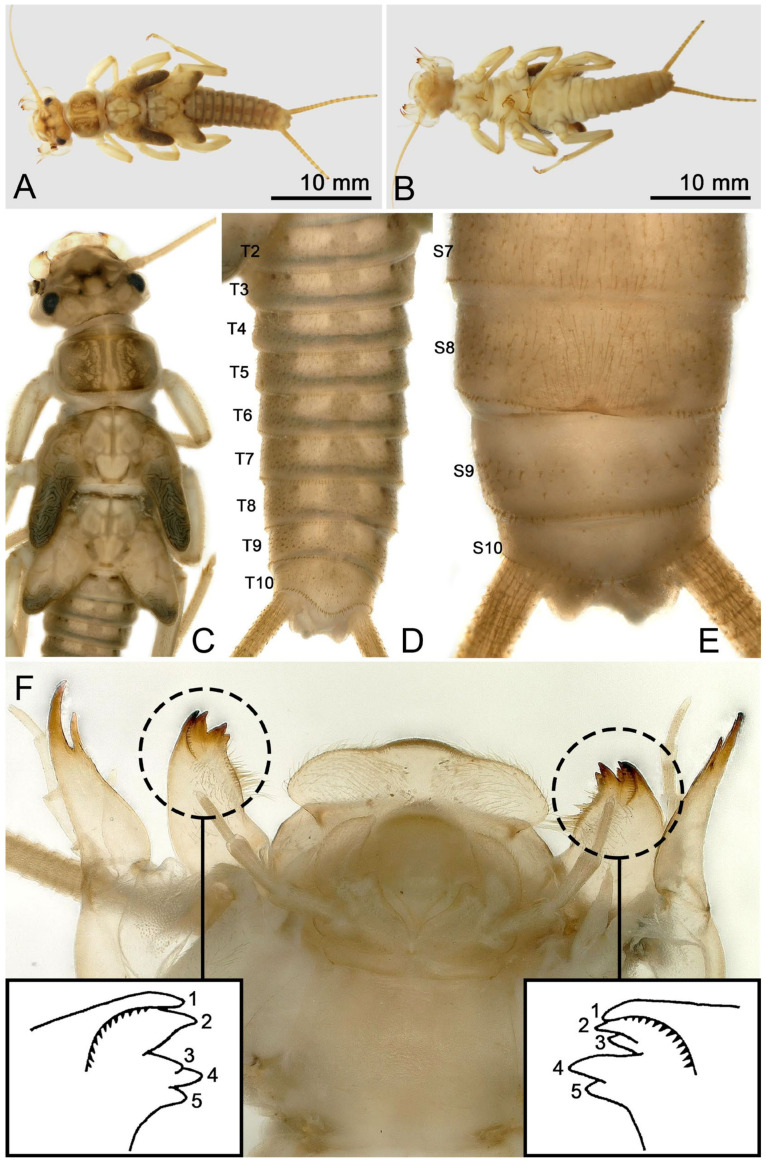
*Perlodinella kozlovi*, female larva from Xinghai (Voucher N1; PV535531). (**A**,**B**) Dorsal and ventral habitus; (**C**) head and thoraxes, dorsal view; (**D**,**E**) abdominal segments, dorsal and ventral views; (**F**) mouthpart with illustrations of the mosaic teeth on right and left mandibles, ventral view.

**Figure 9:**
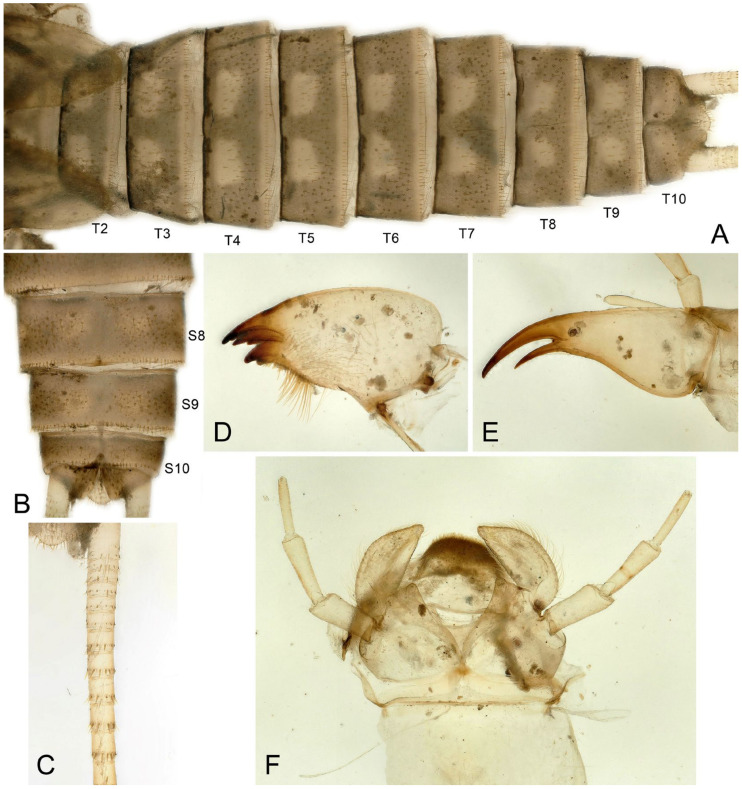
*Perlodinella kozlovi*, female exuviae from Datong, Guanghui Temple. (**A**) Abdominal segments, dorsal view; (**B**) sterna 8–10, ventral view; (**C**) cercus; (**D**,**E**) left mandible and lacinia, ventral views; (**F**) labium.

**Figure 10:**
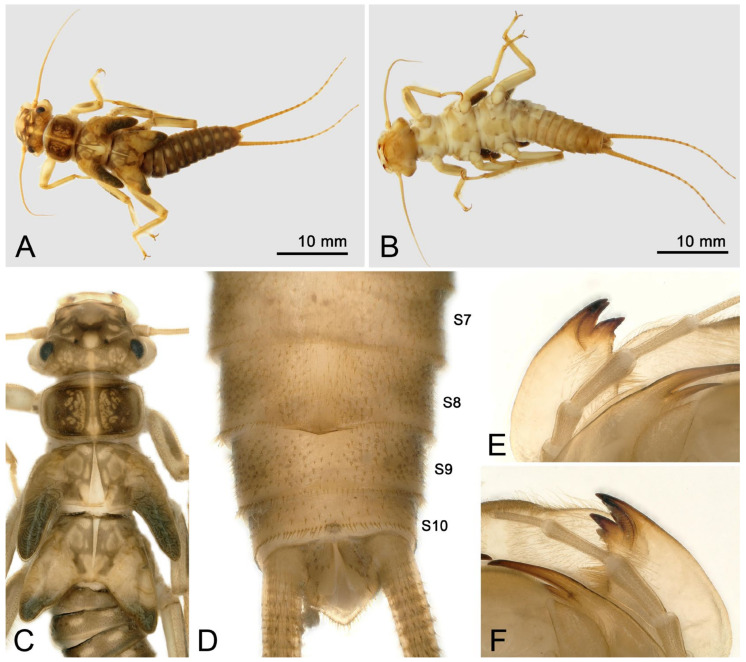
*Perlodinella kozlovi*, female larva from Tongde (Voucher GN3; PV535535). (**A**,**B**) Dorsal and ventral habitus; (**C**) head and thoraxes, dorsal view; (**D**) sterna 7–10; (**E**,**F**) right and left mandible and lacinia, ventral views.

Mature female larvae/exuviae body length 35~41 mm (n = 9). Coloration and morphology similar to male (Figure 8, Figure 9 and Figure 10). Wingpad length regular (Figure 8C). A horizontal, anteromedially concave, triangular sclerite is present on the posterior margin of sternum 8 of the female (Figure 8E, Figure 9B and Figure 10D).

Remarks: The original description and illustration of *P. tatunga* were rather brief, especially the wings were not described by Wu [3], while other diagnoses were also not remarkable enough. The first author once suspected that *P. epiproctalis* was a synonym of *P. tatunga*, but Du mentioned that the type specimens of *P. tatunga* (once disposed and available in the Institute of Zoology, Chinese Academy of Sciences, IZCAS) that he examined [25] were all macropterous, while all the *P. epiproctalis* were macropterous in males. So, the status of *P. tatunga* was not confirmed in Huo et al. [8] and remained a mystery to date.

The type materials of *P. tatunga* by Wu [3] were a holotype male, allotype female, paratype 2 males, 5 females, from Datong County, Guanghui Temple, 10 May 1965, collected by Chou Io; another paratype 1 male, from Xinglongshan, Yuzhong County, Gansu Province, 9 May 1964. We compared the specimens of “*P. tatunga*” from Guanghui Temple with *P. kozlovi* from other counties of Qinghai and finally confirmed that they are the same species based on strict collecting information and morphology of *P. tatunga*.

Additionally, Du [25] mentioned that he ever examined the type specimens of *P. tatunga* preserved at IZCAS and found that the original species name on the label of the type specimens was “周氏罗襀” It is possible that Wu [3] initially intended to name the species after the collector, Prof. Chou Io, using his surname (周 = Zhou or Chou, different romanizations of the same Chinese character).

It was mentioned [9] that there are several variations in the shape of the female subgenital plates of *P. kozlovi* and *P. tibetensis* (from Xizang). The middle posterior margin of the plate may extend a very small protrusion backward, but it may also be concave forward, while only the edges on both sides extend backward. What we are puzzled about is whether the variations in the subgenital plates will be manifested in female larva. Through molecular sequencing and comparisons, we discovered that the unique larva from Tongde (Voucher GN3; PV535535) has the extending subgenital plate, but the color pattern and mouthpart are the same (Figure 10A–F) with other infraspecific larvae we described.

**Figure 11:**
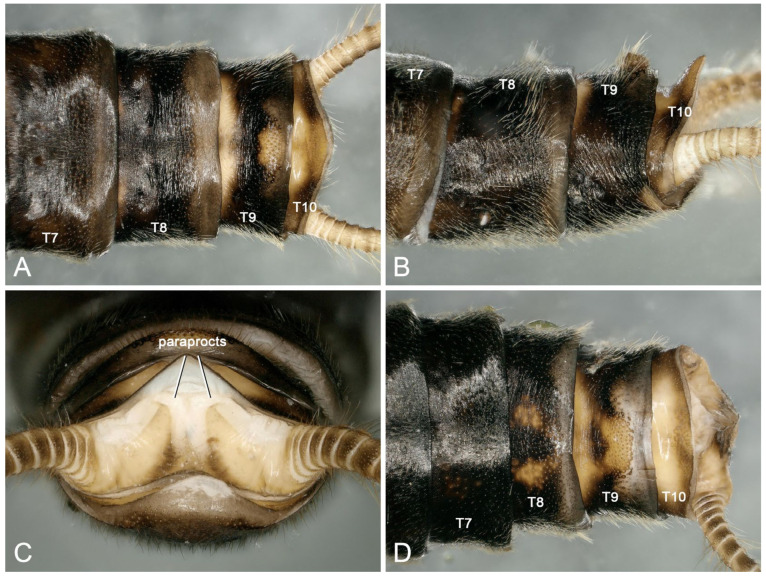
*Perlodinella microlobata*, two males from Tongde. (**A**–**C**) The individual (Voucher GMM; PV535536) with few yellowish patterns, dorsal, lateral and caudal views; (**D**) the individual (Voucher GW; PV535539) with more yellowish patterns on terga 8–10, dorsal view.

**Figure 12:**
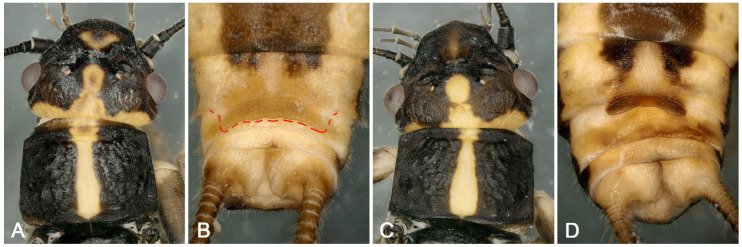
*Perlodinella microlobata* from Tongde, female head, pronotum, and sterna 7–9. (**A**,**B**) Individual with large yellowish frontoclypeal spot and wider subgenital plate; (**C**,**D**) individual (Voucher GMF; PV535537) with few yellowish frontoclypeal spot and more sclerotized subgenital plate.

**Figure 13:**
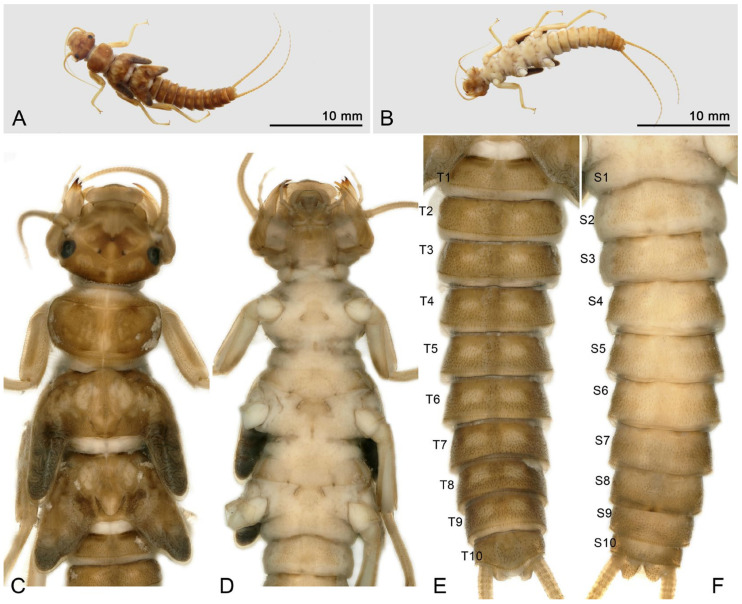
*Perlodinella microlobata*, female larva from Zeku (Voucher N2; PV535538). (**A**,**B**) Dorsal and ventral habitus; (**C**) head and thoraxes, dorsal view; (**D**) sterna 7–10; (**E**,**F**) right and left mandible and lacinia, ventral views.

**Figure 14:**
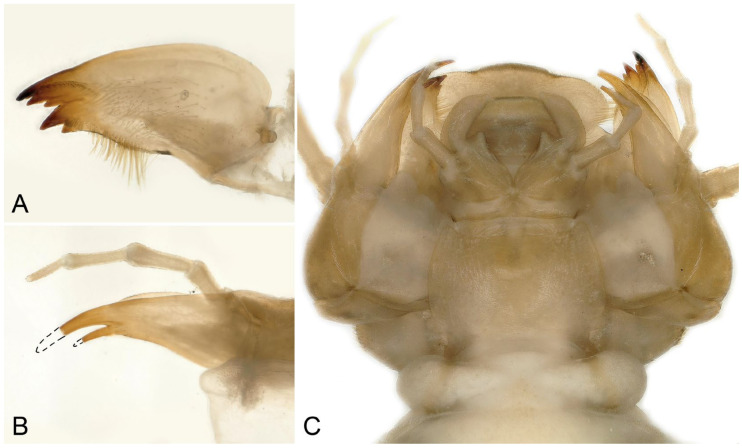
*Perlodinella microlobata*, female larva from Zeku (Voucher N2; PV535538). (**A**,**B**) Left mandible and lacinia, ventral view; (**C**) labium.

***Perlodinella microlobata* Wu, 1938 (Figure 11, Figure 12, Figure 13 and Figure 14)**

*Perlodinella microlobata* Wu, 1938: 55.

Material examined: 3 males, 2 females, 1 larvae, China, Qinghai Province, Hainan Tibetan Autonomous Prefecture, Tongde County, Zhihoumai Village, Gagan Reservoir, 2024-VI-4, 35.123291° N, 100.622657° E, 3347 m. Leg. Huo Qing-Bo, Fan Shi-Xiong. The same collection sites were recorded in Huo et al. [10] 1 male, China, Qinghai Province, Haixi Mongol and Tibetan Autonomous Prefecture, Tianjun County, 2021-VII-13, 37.568689° N, 98.657256° E, 3545 m, leg. Huo Qing-Bo; 1 larva, Qinghai Province, Huangnan Tibetan Autonomous Prefecture, Zeku County, Heri Town, G573 highway, 2024-VI-5, 35.256546° N, 101.050529° E, 3465 m. Leg. Huo Qing-Bo, Fan Shi-Xiong. 1 male, 3 females, China, Sichuan Province, Garze Tibetan Autonomous Prefecture, Shiqu County, 2009-VI-29, 32.838611° N, 98.376944° E, 4200 m, leg. Qian Yu-Han. All specimens disposed in ICYZU.

Distribution: China: Qinghai (Tianjun, Tongde, Zeku counties), Sichuan (Shiqu County). The distributions in northern Gansu (locality unclear), Shanxi, and Liaoning provinces are unconfirmed because of a lack of an available specimen for examination and recent field observation.

Supplementary description of adults: Male individuals of this species have yellow spots of variable shapes and sizes in the middle of terga 8–9. The area surrounding the conical sensilla on T9 is yellow, while the surrounding regions are mostly black. However, the color of the anterior and posterior edges of T9, extending toward the conical sensilla, may also be lighter (Figure 11A–D).

The posterior edge of female subgenital plate is concave anteriorly, may be strongly sclerotized, and extends posteriorly into a small protrusion (Figure 12A–D). The morphology of females from Qinghai is consistent with that of the population from Sichuan described by Huo et al. [10] and is unique within this genus. Therefore, it can serve as a reliable basis for field identification.

Larva: Male larvae/exuviae unknown. Female larva: body length 20 mm (n = 1). All the morphology of mouthpart and subgenital plate similar to *P. kozlovi* (Figure 13A–F and Figure 14A–C). The remarkable diagnosis of the larva is that the coloration is darker than *P. kozlovi* and with a few pale patterns on the head and thoraxes (Figure 13C); the seta on abdominal terga are much thicker and denser (Figure 13E) than *P. kozlovi* (by comparison with the larvae and exuviates, Figure 5, Figure 6, Figure 7, Figure 8, Figure 9 and Figure 10).

**Figure 15:**
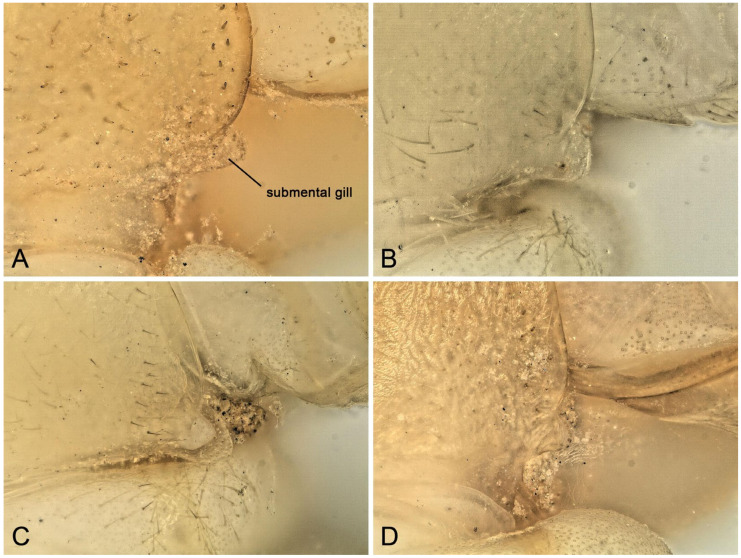
Submental gills of *Perlodinella* larvae, High Dynamic Range versions. (**A**) Male larva of *P. epiproctalis* from Qilian Mountains; (**B**) female larva of *P. kozlovi* from Xinghai (Voucher N1; PV535531); (**C**) female larva of *P. kozlovi* from Tongde (Voucher GN3; PV535535); (**D**) female larva of *P. microlobata* from Zeku (Voucher N2; PV535538).

Remarks: The submental gills of the larvae of the above three species are short, membranous protuberances, usually with cuticular asperities on the apex (Figure 15A–D). In previous studies, the gill of *Perlodinella* was not well described [8,9]. The materials that we examined indicate that the gill’s morphology did not change significantly among the three species (whether in different geographical populations or genders) and thus could be typical in the genus.

***Perlodinella unimacula* Klapálek, 1912 (nomen dubium)**

*Perlodinella unimacula* Klapálek, 1912: 30.

*Perlodes (Perlodinella) unimacula*: Brinck, 1954: 198.

*Skobeleva unimacula*: Wu, 1973: 98.

Distribution: China: Qinghai (Zadoi County).

Remarks: The original record of the type locality for this species is “Oberlauf des Chi-Tschu, Bassin des Blauen Flusses. Ges. von Kozlov, Mitte Juli 1900. (Mus. St. Petersburg).” Wu [2] translated these records into English: “TYPES: Two females from upper course of Chi-Tschu in the basin of Blue River; July 1900, collected by Kozlov. In Mus. St. Petersburg.” Later, DeWalt et al. [12] provided a corrected geographical location: “China, Qinghai Province, Yushu Tibetan Autonomous Prefecture, upper reaches of I Chu River, a tributary of Changjiang River in the Bayan Har Mts”. Based on our investigation, this area likely refers to the Yiqu River basin, located between Zhiduo County and Zadoi County in Qinghai, which is a tributary of the Changjiang River in the Bayan Har Mountains.

**Figure 16:**
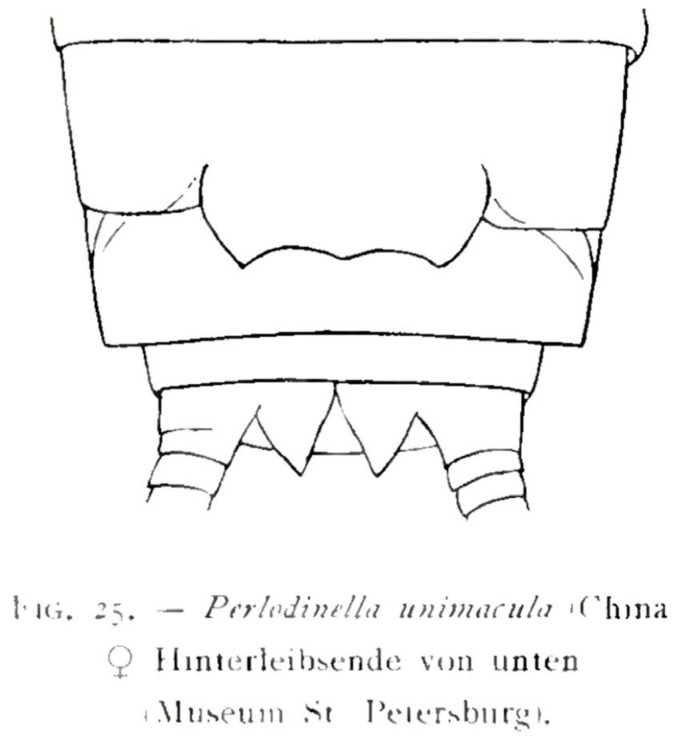
The terminalia of *Perlodinella unimacula*, illustrated by Klapálek [1].

Considering that the morphological description of *P. unimacula* is overly simplistic and vague, as well as the extensive intraspecific morphological variations in the genus, it is no longer possible to distinguish this species from others merely by the outline of the female (Figure 16). Geographically, the distribution position of *P. unimacula* highly coincides with that of *P. kozlovi* and *P. microlobata*, and it is very likely to be synonymic of one of the latter. Therefore, we consider that *P. unimacula* should be specified as nomen dubim.

### 3.3. Biological Adaptations in Natural and Artificial Landscapes

**Figure 17:**
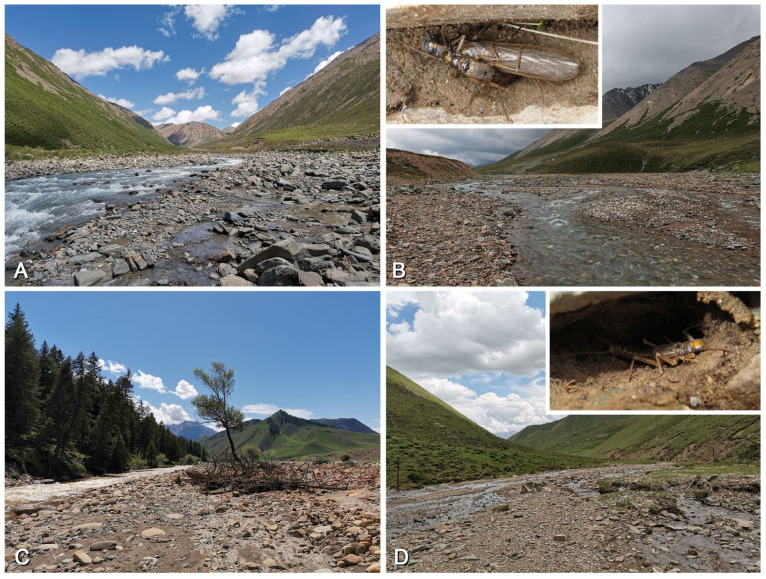
Collecting sites and habitat of *Perlodinella epiproctalis*, with the adults observed sheltering within caves. (**A**,**B**) Menyuan, 3720 m; (**C**,**D**) Qilian, 3122–3425 m.

The habitat environments of *Perlodinella epiproctalis*, *P. kozlovi*, and *P. microlobata* each exhibit certain remarkable characteristics. Based on our observations, populations of *Perlodinella epiproctalis* appear to be restricted to high-altitude areas (above 3500 m) in the Qilian Mountains, particularly in the Datong River basin (Figure 17A–D). The species inhabits wide but shallow streams and is currently the largest sized perlodid in Qinghai. In streams where *P. epiproctalis* is present, almost no other Perlodidae species have been found, which implies that it could be the dominant species there. The adults of *P. epiproctalis* exhibit significant negative phototaxis. They prefer to gather under stones for mating and quickly retreat into crevices when exposed to strong light. In this study, we observed that some males often have thick layers of mud covering their bodies and hide in smooth-walled mud chambers (Figure 17B). While this does not confirm that they have burrowing habits, we do not rule out the possibility that this species may utilize nests left by other soil-dwelling animals as shelters.

**Figure 18:**
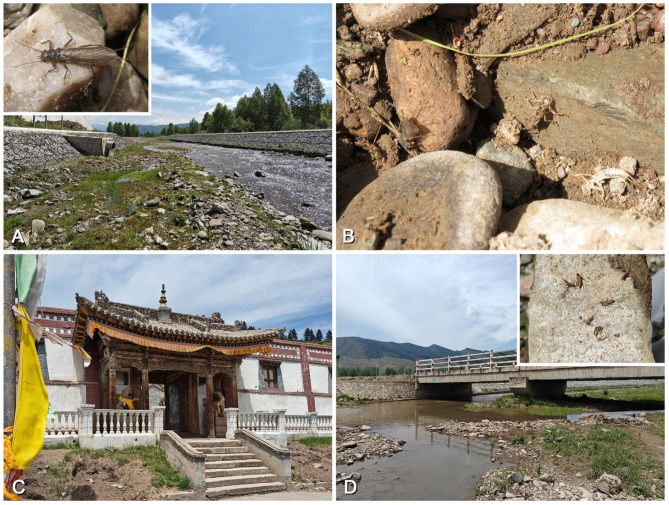
Collecting sites and habitat of *Perlodinella kozlovi* in Datong, with the adults or exuviae observed under the rocks. (**A**,**B**) The river 5 km south of Guanghui Temple, 2575 m, riverbank has been artificially reinforced; (**C**) Guanghui temple, originally constructed in the 17th century; (**D**) the river 1.5 km north of Guanghui Temple, flowing through the town.

**Figure 19:**
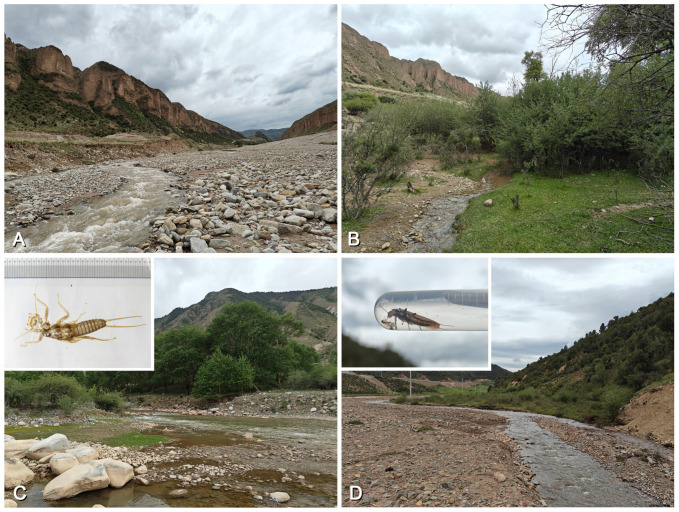
Collecting sites and habitat of *Perlodinella kozlovi*, with different altitudes and a lack of human activities. (**A**,**B**) Xinghai, 3004 m, larva only; (**C**,**D**) Tongren, 2675 m, female adult and exuviae larva only.

**Figure 20:**
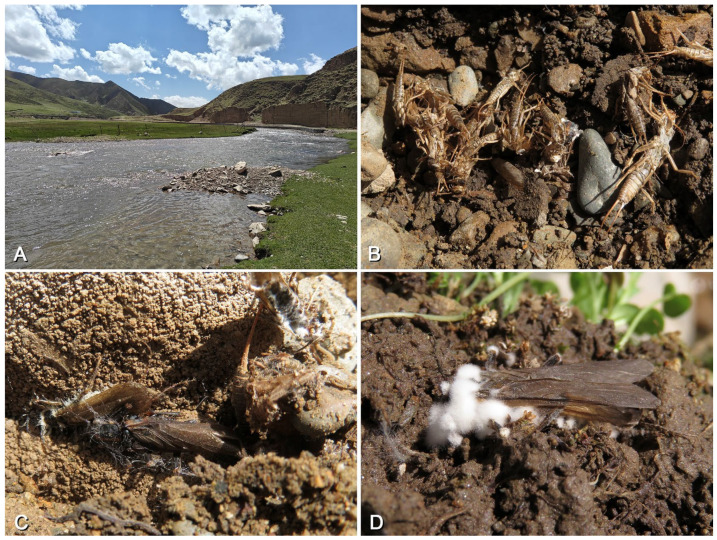
Collecting sites and habitat of *Perlodinella kozlovi* and *P. microlobata* in Tongde. (**A**) The river beside Gagan Reservoir, 3347 m; (**B**) multiple exuviae; (**C**,**D**) the *Perlodinella* adults who were infected with fungi and died beside the exuviae.

*Perlodinella kozlovi* is already the most widely distributed and urban-adapted perlodid species in Qinghai, primarily recorded in streams near cities at elevations of 1500 to 2500 m, but also found at 3500 m (Figure 18, Figure 19 and Figure 20). In recent years, the Qinghai Provincial Government has been committed to addressing ecological issues caused by overgrazing and environmental pollution, particularly in the Three-River-Source region and around Qinghai Lake (Central People’s Government, https://www.gov.cn/xinwen/2014-04/18/content_2662332.htm; Qinghai Daily, https://baijiahao.baidu.com/s?id=1795163307590330355&wfr=spider&for=pc; accessed on 20 March 2025). Improvements in vegetation conditions and reductions in pollution sources have further alleviated the survival threats faced by local stoneflies, and then, *P. kozlovi* and some other aquatic creatures could be able to survive the process of urbanization. In river sections flowing through suburban areas (even those with artificially hardened banks), *P. kozlovi* is still able to grow and emerge naturally (Figure 18A–D). In most cases, both natural and artificial riverbanks are dominated by rocks and grass, with a lack of shrubs and trees. It should be noted that the emergence timing of *P. kozlovi* populations varies across different altitudes: in Tianjun (altitude 3545 m), many adults can still be collected in early July, while at Datong, Tongde counties, etc., with lower altitudes (altitude 2500~3000 m) and higher temperatures, more exuviae or females than males can be found by late May (Figure 18 and Figure 19), and no adults were observed in our surveys after July.

*Perlodinella microlobata* is the most mysterious perlodid species in Qinghai. Few have been observed in recent years, but they are often found coexisting with *P. kozlovi* populations, both in Tianjun and Tongde counties (Figure 20A). Similar to *P. epiproctalis*, the adults of *P. microlobata* and *P. kozlovi* also prefer to be active under rocks, the wall roots of cement bridges, or other buildings along the river and exhibit negative phototaxis (which makes the *Perlodinella* playfully called “the wallfacers” by collectors). However, we once observed a female *P. kozlovi* crawling on a shrub in direct sunlight (Figure 19D), possibly because it needed to reach a nearby stream to lay eggs.

### 3.4. The Threat from Fungal Infections

Currently, no animals specialized in preying on stoneflies have been reported in Qinghai. Given that adult *Perlodinella* mostly inhabit areas under rocks and the streams where larvae live lack large benthic invertebrates (e.g., crabs) and fish, the threat that they face from aquatic predators is likely minimal. However, the threat to the population of stoneflies comes more from the microorganism. Over the past three years, we found that *Perlodinella kozlovi* is susceptible to fungal infections (Figure 20A–D). Since the larvae of *P. kozlovi* emerge on land (Figure 20A) and under the rocks, fungal spores can spread rapidly under conditions of a high host population density (10–50 adults/m^2^) and result in the death of these adults in situ (Figure 20C–D). A notable phenomenon is that many *P. kozlovi* adults die near their exuviae and eventually develop visible white fungal hyphae, which implies that they died shortly after emergence rather than due to normal aging.

**Figure 21:**
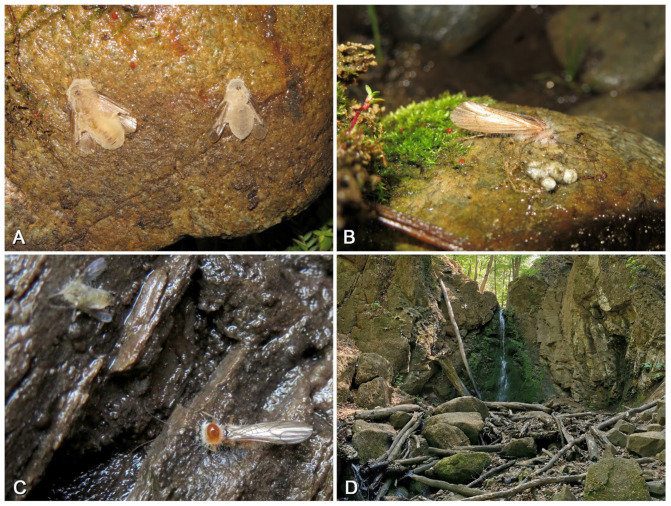
The similar fungal infection in our other observations. (**A**,**B**) The Trichoptera species from Qilian, 3424 m, 6 July 2021; (**C**,**D**) the Nemouridae and Diptera species (**C**) in a waterfall near Parád, Hungary (**D**), 458 m, 12 May 2022.

This is the first report of stoneflies in China being parasitized and killed by fungi. This indicates that stoneflies seem lack effective defense mechanisms against microbial attacks, and their population sizes may be significantly regulated by infection and mortality rates. These cases are currently sporadic but not unique to the stonefly or to China. The first author has also observed similar infections in caddisflies (Trichoptera) in Qinghai (Figure 21A,B) and another insect fauna in Europe, including Nemouridae and some Diptera (Figure 21C,D). Previous research has paid little attention to the interactions between microorganisms and stoneflies [26,27,28]. In terms of more macroscopic population influencing factors, the survival threats faced by stoneflies are mostly attributed to climate change, environmental pollution, or habitat destruction [29,30], but there is a lack of direct evidence from microbial influences. Our observation records may provide a new idea and perspective for future ecological work.

### 3.5. The Distribution, Origin, and Immigration

**Figure 22:**
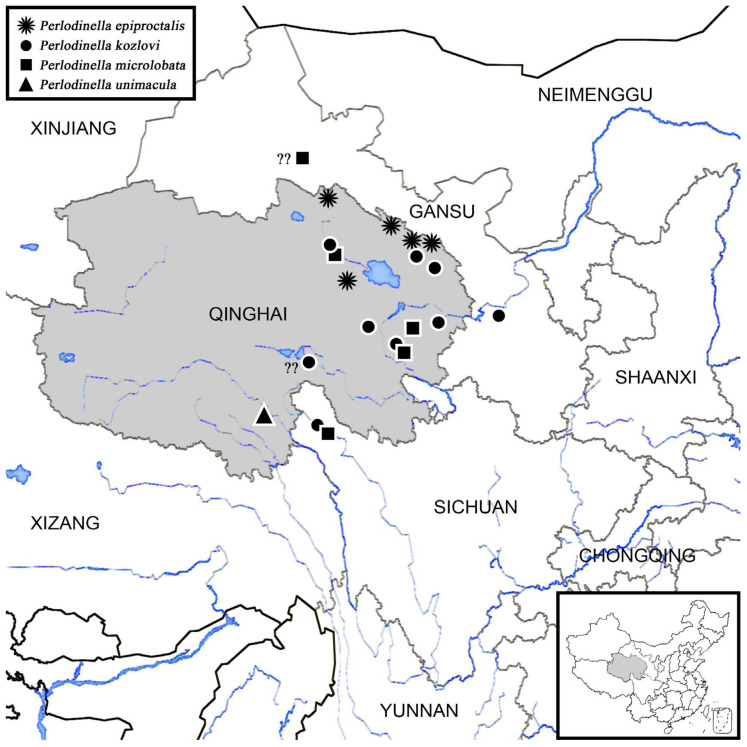
The distribution of *Perlodinella* in Qinghai and neighboring provinces with the main water systems (marked in blue). The position of Qinghai in China is shown in the lower right corner, and the doubtful/unclear localities that without exact longitude and latitude are marked with “??”. Map revised using www.tianditu.gov.cn, accessed on 10 February 2025.

**Figure 23:**
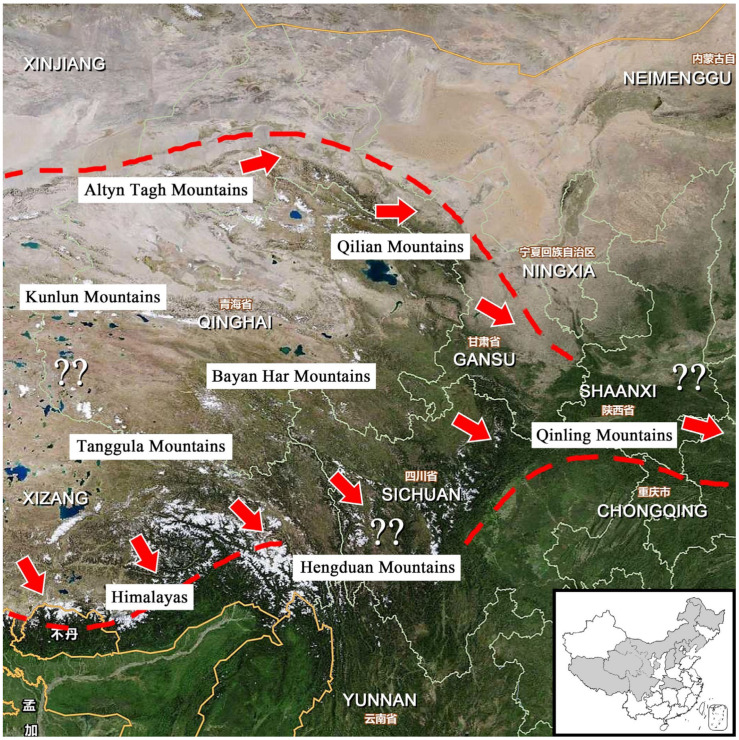
The hypothetical immigration of *Perlodinella* around Qinghai (the migrating direction of populations is marked with arrows; the potential distribution areas are marked by “??”; the dashed line indicates the potential boundary for population dispersal), and the distribution of *Perlodinella* in China is shown in the lower right corner. Map revised using www.tianditu.gov.cn, accessed on 10 February 2025.

The three species of *Perlodinella* in Qinghai are primarily distributed in the eastern part of the province, with occasional reports from neighboring Gansu and Sichuan provinces, i.e., northern and eastern parts of the Tibetan Plateau (Figure 22). To date, no records of *Perlodinella* have been found in the Xinjiang Uygur Autonomous Region, Mongolia, Bhutan, or India, etc., which are near the Qinghai-Tibet Plateau. Qinghai contains numerous rivers, streams, and salt lakes, including the sources of the three major water systems of the Changjiang River, the Yellow River, and the Pearl River (famous as the Three-River Source Region, TRSR, or Sanjiangyuan). Theoretically, the mountains (Mts. Kunlun and Altyn Tagh) western to TRSR from western Qinghai to southeastern Xinjiang could also be potential distribution areas for *Perlodinella*. However, many regions in these areas are dry and lack freshwater resources (Figure 23), making them seemingly unsuitable for stonefly survival. The mountains in Sichuan, Chongqing, and Yunnan that are southeast to Qinghai can also provide a potential habitat for *Perlodinella*, but the genus has not been found based on the relatively high sampling rates there so far. Therefore, the western and northern regions of the Qinghai-Tibet Plateau likely represent the origin and speciation center for this genus.

Considering that the origin and dispersal of stoneflies are closely tied to the direction of river systems [19,20], but water systems are also influenced by mountains and altitude [18], the limited distribution of *Perlodinella* may be following three hypotheses:

(1) The southward expansion of the population is obstructed by mountain ranges, while its northward dispersal is hindered by deserts or arid regions: *Perlodinella* may not have the ability to cross the higher-altitude Himalayan and Hengduan Mountains to spread southward. Additionally, the regions north and west of the Altyn Tagh-Qilian Mountains, being close to deserts and lacking streams, have prevented *Perlodinella* from establishing populations further north (though they may have once inhabited these areas). This suggests that the genus likely originated after the formation of these mountains.
(2) Environmental-change-induced niche mismatch: In Qinghai, *Perlodinella* inhabits flat, slow-moving rivers that lack predators and riparian vegetation. However, as the latitude and altitude decrease, accompanied by rising temperatures and increasing biodiversity, such habitats and ecosystems become rare in provinces west of Qinghai. Based on our sampling experience, from southeastern Xizang to central/south China, the typical mountainous landscapes predominantly consist of dense forests, heavily shaded river valleys, cascading waterfalls, and more diverse aquatic predators (including natural enemies and aquatic competitors occupying similar ecological niches, at least multiple genera of Perlidae and other Perlodidae).
(3) The lineage within *Perlodinella* may have dispersed through the Qinling Mountains and finally settled in the northeastern China: Interestingly, in *Perlodinella*, there is still one species recorded from central China (*P. shennongjia* Chen, Xu & Shen, 2022, from Hubei Province [31]), and the other two species were recorded in northeastern China (*P. mazehaoi* Chen, 2019 from eastern Inner Mongolia [7]; *P. fuliginosa* Wu, 1973 from western Heilongjiang Province).

Based on the above observations, *Perlodinella* appears to be largely constrained from further expansion into other areas that are more northern, western, or southern than Qinghai. Thus, Qinling Mountains east of the Qilian range likely served as the sole biogeographic corridor enabling this genus to penetrate central China and ultimately reach northeastern regions. Qinling is widely recognized as both the dividing line between northern and southern China and the transitional zone between the Palaearctic and Oriental realms. This mountain barrier correlates with the markedly reduced species diversity and distribution records of Holarctic stoneflies (particularly Capniidae, Perlodidae, and Chloroperlidae) in areas south of Qinling [11,25,32,33]. The future biogeographic studies on multispecies may provide stronger evidence supporting the dispersal pattern that we proposed. Such research could help determine whether the northeastward expansion of *Perlodinella* represents a unique case or reflects broader distributional trends among the stonefly taxa of China.

## Figures and Tables

**Table 1:** Sample information with GenBank accession numbers of the COI sequences of *Perlodinella* species from Qinghai.

No.	Species Name	Voucher	Accession Number	Locality	Collecting Date	Coord	Sex	Stage
1	*P. epiproctalis*	E3	PV535523	Menyuan County	4 July 2021	101.582778° E, 37.585556° N	Male	Adult
2		E5	PV535522	Menyuan County	4 July 2021	101.582778° E, 37.585556° N	Female	Adult
3		E6	PV535521	Menyuan County	4 July 2021	101.582778° E, 37.585556° N	Female	Adult
4	*P. kozlovi*	k1	PV535532	Tianjun County	13 July 2021	37.56869° N, 98.65726° E	Male	Adult
5		K2	PV535529	Tianjun County	13 July 2021	37.56869° N, 98.65726° E	Female	Adult
6		K4	PV535528	Tianjun County	13 July 2021	37.56869° N, 98.65726° E	Female	Adult
8		DKF	PV535530	Datong County	27 May 2024	37.023000° N, 101.783832° E	Female	Adult
9		M	PV535527	Datong County	27 May 2024	37.023000° N, 101.783832° E	Male	Adult
10		PKM	PV535525	Datong County	27 May 2024	37.023000° N, 101.783832° E	Male	Adult
11		PKF	PV535534	Datong County	27 May 2024	37.023000° N, 101.783832° E	Female	Adult
12		GN3	PV535535	Tongde County	4 June 2024	35.123291° N, 100.622657° E	Female	Larva
13		PM	PV535524	Tongde County	4 June 2024	35.123291° N, 100.622657° E	Male	Adult
14		PF	PV535526	Tongde County	4 June 2024	35.123291° N, 100.622657° E	Female	Adult
15		N1	PV535531	Xinghai County	30 May 2024	35.581647° N, 99.863318° E	Female	Larva
16	*P. microlobata*	GMM	PV535536	Tongde County	4 June 2024	35.123291° N, 100.622657° E	Male	Adult
17		GMF	PV535537	Tongde County	4 June 2024	35.123291° N, 100.622657° E	Female	Adult
18		GW	PV535539	Tongde County	4 June 2024	35.123291° N, 100.622657° E	Male	Adult
19		N2	PV535538	Zeku County	5 June 2024	35.256546° N, 101.050529° E	Female	Larva

## Data Availability

The data that support the findings of this study are available in NCBI: GenBank accession nos. of mitogenomes: PV535521-PV535532, PV535534-PV535539.

## Supplementary Materials

The following supporting information can be downloaded at: https://www.mdpi.com/article/10.3390/insects16050520/s1, Table S1 generic distance MEGA-result1. Reference [24] is cited in the Supplementary Materials.
